# Systematic review and meta-analysis comparing postoperative and oncological outcomes between local excision plus radiotherapy versus total mesorectal excision for rectal cancer

**DOI:** 10.1186/s12957-026-04304-8

**Published:** 2026-04-02

**Authors:** Daichi Kitaguchi, Antonello Forgione, Chiara Innocenzi, Yuchuan Yang, Federico Espínola, Mariano Giménez, Tatsuya Oda, Jacques Marescaux

**Affiliations:** 1 Research Institute against Digestive Cancer (IRCAD), IRCAD France, 1 place de l’Hôpital, Strasbourg, 67091 Strasbourg Cedex France; 2 Department of Gastrointestinal and Hepatobiliary and Pancreatic Surgery, Institute of Medicine, University of Tsukuba, Ibaraki, Japan; 3 Department of Women, Children, and Public Health Sciences, Fondazione Policlinico Universitario A Gemelli IRCCS, Rome, Italy

**Keywords:** Rectal Neoplasms, Transanal Endoscopic Microsurgery, Proctectomy, Colorectal Surgery

## Abstract

**Background:**

Total mesorectal excision (TME) is the standard treatment for middle to lower rectal cancer. However, it is associated with significant postoperative complications and functional impairments. Local excision (LE) combined with neoadjuvant radiotherapy (RT) has emerged as a less invasive alternative and has shown potential for organ preservation. This study aimed to compare the short-term and long-term outcomes of LE + RT versus TME in selected patients with rectal cancer.

**Materials and methods:**

This systematic review and meta-analysis were conducted following a comprehensive literature search of human studies published in English, completed by April 2025. Inclusion criteria consisted of rectal cancer up to cT3 disease and/or showed a favorable response to neoadjuvant therapy. Postoperative outcomes assessed included operative time, blood loss, morbidity, length of hospital stay, and permanent stoma rate. Oncological and survival outcomes were also evaluated.

**Results:**

A total of six studies were included. They consisted of five randomized controlled trials and one prospective clinical trial, with a total of 679 patients. Of these, 366 were treated with LE + RT and 313 with TME. Pooled analysis showed that the LE + RT group was associated with a significantly shorter operative time, a lower overall morbidity, and a shorter postoperative hospital stay. No significant differences were observed between the groups in oncological or survival outcomes.

**Conclusions:**

This study suggests that, in selected rectal cancer patients, LE + RT may represent a less invasive alternative with comparable oncological outcomes to TME. LE + RT may subsequently be a viable treatment option, especially in patients seeking organ preservation or in patients unfit for TME.

**Supplementary Information:**

The online version contains supplementary material available at 10.1186/s12957-026-04304-8.

## Introduction

The standard treatment for middle to lower rectal cancer is total mesorectal excision (TME), with or without neoadjuvant chemoradiotherapy (CRT) [1, 2]. However, surgery is associated with significant complications, with early or late morbidity reported in 25 to 50% of patients [3–5]. Additionally, even among cancer survivors, long-term bowel, bladder, and sexual dysfunctions following TME often impair quality of life (QoL) [6, 7]. To prevent such postoperative adverse events, nonoperative management such as the watch-and-wait (WW) strategy has emerged as a potential alternative strategy for locally advanced rectal cancer in patients who achieve a clinical complete response (cCR) or near complete response (nCR) on reassessment after neoadjuvant RT [8–10]. However, key challenges associated with WW include the diagnostic difficulty and uncertainty in distinguishing cCR, nCR, and incomplete response (iCR) [11], as well as the relatively high incidence of tumor regrowth during surveillance, even among patients initially assessed as having achieved cCR or nCR [12–14].

Local excision (LE), particularly transanal endoscopic microsurgery (TEM), offers a high-quality, sphincter-preserving approach to rectal cancer treatment [15]. When combined with neoadjuvant RT, such as long-course CRT or short-course RT (SCRT), LE may allow for a more accurate evaluation of CR through pathological assessment. It may also contribute to enhance the local control effect of neoadjuvant treatment alone, thereby reducing the risk of local regrowth. The combination of neoadjuvant RT, with LE may serve as a potential strategy to bridge the paradox in the treatment of rectal cancer. Several clinical trials comparing LE combined with neoadjuvant RT versus TME in selected patients with rectal cancer were reported. However, no definitive conclusions were reached, and the combination of neoadjuvant RT with LE has still not been widely accepted. This ongoing uncertainty drove us to conduct a systematic review and meta-analysis.

The objective of this study was to compare the short-term postoperative outcomes and long-term oncological outcomes of LE combined with neoadjuvant RT, versus TME in rectal cancer patients with clinical staging up to cT3 and/or a favorable response after neoadjuvant therapy. Additionally, the study sought to clarify the risks and benefits of LE combined with neoadjuvant RT, as a potential surgical alternative to TME in such selected cases. This updated meta-analysis only included prospective studies and incorporated the most recent long-term outcomes, providing more comprehensive and up-to-date evidence.

## Materials and methods

### Literature search

This systematic review was conducted in accordance with the Preferred Reporting Items for Systematic Reviews and Meta-Analyses (PRISMA) guidelines [16]. A prospective protocol was registered with the International Prospective Register of Systematic Reviews (PROSPERO) under registration number CRD420251048717. The full record is available at: https://www.crd.york.ac.uk/PROSPERO/view/CRD420251048717. A comprehensive literature search was performed using PubMed, Embase, and the Cochrane Library. A core search algorithm consisting of the terms (rectal neoplasms) AND (local excision) AND (total mesorectal excision) was developed. This strategy was adapted for each database using appropriate controlled vocabulary (e.g., MeSH and Emtree) and database-specific syntax. The complete search strategies are provided in Supplementary Table 1. The results were limited to human studies published in English, and the final search was completed by April 2025.

### Inclusion and exclusion criteria

Articles were considered eligible if they met the following criteria:

1. Eligible studies were prospective comparative clinical trials, including randomized controlled trials (RCTs) and non-randomized prospective comparative studies;
2. The study population consisted of patients with rectal cancer who had up to cT3 disease and/or showed a favorable response to neoadjuvant therapy;
3. Patients in the LE group underwent neoadjuvant RT (either long-course CRT or SCRT) although studies were included regardless of whether patients in the TME group received neoadjuvant therapy;
4. The study evaluated the efficacy and/or safety of LE vs. TME;
5. At least one postoperative outcome or oncological outcome was reported;
6. Sufficient data were provided to calculate risk ratios (RR) and mean differences (MD).

The following criteria were excluded: study protocols, unpublished studies, non-original articles (including letters, comments, abstracts, corrections, and replies), retrospective studies, studies lacking sufficient data, and review articles.

### Data extraction

Data extraction was independently performed by two authors, and any discrepancies were resolved through discussion. All articles identified in the initial search were manually and independently screened based on their titles and abstracts, following the predefined eligibility criteria. Articles that passed the initial screening underwent full-text review to determine final inclusion. Postoperative outcomes included operative time, intraoperative blood loss, overall morbidity (including all Clavien–Dindo grades), severe morbidity (defined as Clavien–Dindo grade ≥ 3), postoperative hospital stay, and permanent stoma rate. Oncological outcomes included positive margin rate, local recurrence rate, and overall recurrence rate, which encompassed both local and distant recurrences. When multiple publications reported outcomes from the same patient cohort, only the most recent and comprehensive dataset was included in the quantitative meta-analysis to avoid double counting.

### Quality assessment

All RCTs were assessed for risk of bias using the revised Cochrane tool for randomized trials (RoB 2) [17], whereas non-randomized clinical trials were evaluated using the Risk of Bias in Non-randomized Studies of Interventions tool (ROBINS-I) [18]. Two authors assessed the quality of all included studies, resolving any disagreements through discussion.

### Statistical analysis

Meta-analyses were conducted using random-effects models and presented as forest plots. For dichotomous outcomes, pooled estimates were calculated using the Mantel-Haenszel method, with between-study variance estimated using the DerSimonian-Laird approach, and were expressed as risk ratios (RRs) with 95% confidence intervals (CIs). For continuous outcomes, pooled estimates were calculated using the inverse-variance method with between-study variance estimated using Restricted Maximum Likelihood (REML). Confidence intervals were constructed using the Hartung-Knapp-Sidik-Jonkman (HKSJ) adjustment, and results were expressed as mean differences (MDs) with 95% CIs. For time-to-event outcomes, including overall survival (OS) and disease-free survival (DFS), log-transformed hazard ratios (HRs) and their standard errors (SEs) were synthesized using REML. Corresponding 95% CIs were calculated using the HKSJ adjustment. Statistical heterogeneity was assessed using Cochran’s Q test (Chi² test) and quantified using the inconsistency index (I²). A Chi² P-value < 0.10 or an I² value > 50% was considered indicative of substantial heterogeneity. For continuous outcomes reported as medians with ranges or interquartile ranges (IQRs), means and standard deviations (SDs) were estimated using the methods described by Wan et al. and Luo et al. [19, 20]. All meta-analyses were performed using RevMan Web (Cochrane, London, United Kingdom).

## Results

### Study selection

**Fig. 1:**
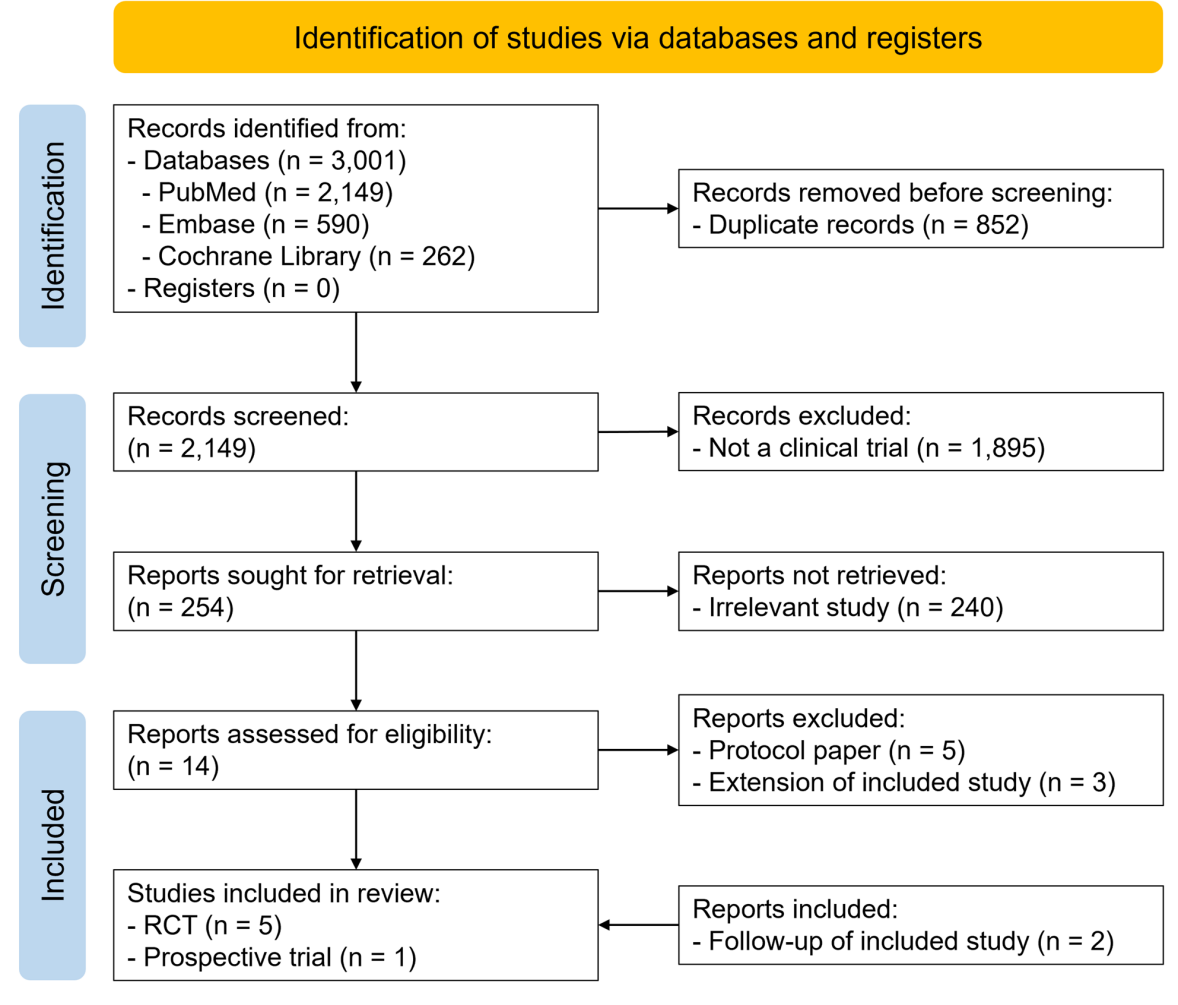
PRISMA flow diagram

The PRISMA flow diagram is shown in Fig. 1. A total of 3,001 records were identified through database searches, including 2,149 from PubMed, 590 from Embase, and 262 from the Cochrane Library. After removing 852 duplicate records, 2,149 unique records remained. Of these, 1,895 were excluded as they were not clinical trials, leaving 254 reports for title and abstract screening. During this process, 240 reports were excluded due to irrelevance, and 14 reports were selected for full-text screening. Among 14 full-text reports, five were excluded as study protocols, and three were excluded as extension or follow-up reports of already included studies. Ultimately, six studies met the eligibility criteria for inclusion in this review, comprising five RCTs [21–25] and one prospective clinical trial [26]. A follow-up study reporting the 5-year results of one of the previously included RCTs was identified [27], and an updated search conducted in May 2025 identified another follow-up study reporting the long-term results of the other previously included RCT [28]. Two of the included RCTs [21, 22] were conducted by the same research group and appeared to include partially overlapping patient populations. Only the more recent and comprehensive dataset was included in the quantitative meta-analysis to avoid potential double counting.

### Study characteristics

The included studies consist of five RCTs and one prospective clinical trial. In total, 679 patients were enrolled. Of these, 366 patients underwent CRT or SCRT, followed by LE, primarily TEM, and were assigned to the LE + RT group. The remaining 313 patients underwent TME; some received CRT followed by TME, whereas others underwent upfront TME, and were classified into the TME group. The inclusion criteria primarily consisted of tumors up to cT3, cN0 status, and a tumor size of less than 3 to 4 cm. Two studies allowed the inclusion of patients with cT4 or cN+ disease. However, these patients were enrolled only if they had achieved a cCR or nCR after CRT. The study characteristics, including patient details and intervention contents, are presented in Table 1, and the clinical and pathological tumor stages are presented in Table 2.

**Table 1:** Summary of study characteristics, including patient demographics and details of interventions

Author and year (country)	Design (No. of centers)	Sample size (LE vs. TME)	Patients	Interventions	Comparison	Outcomes	Follow-up (median, month)
Bach 2021 (UK)	RCT and prospective (21)	RCT: 27 vs. 28 Prospective: 61 vs. 7	T1-2 N0 Size ≤3 cm	SCRT (25 Gy) followed by TEM	Upfront TME	Short- and long-term	RCT: 51.4 Prospective: 48.8
Lezoche 2008 (Italy)	RCT (2)	35 vs. 35	T2 N0 Grade 1–2 Size <3 cm AV ≤6 cm	CRT (50.4 Gy) followed by TEM	CRT (50.4 Gy) followed by TME	Short- and long-term	84
Lezoche 2012 (Italy)	RCT (2)	50 vs. 50	T2 N0 Grade 1–2 Size <3 cm AV ≤6 cm	CRT (50.4 Gy) followed by TEM	CRT (50.4 Gy) followed by TME	Short- and long-term	115.2
Qiu 2025 (China)	Prospective (6)	38 vs. 41	T3-4 N0 or Tany N+ AV ≤ 10 cm cCR or near cCR	CRT (45–50 Gy) followed by TEM	CRT 45–50 Gy followed by TME	Short- and long-term	60
Rullier 2017/2020 (France)	RCT (15)	74 vs. 71	T2-3 N0-1 AV ≤8 cm Size ≤4 cm Scar ≤2 cm	CRT (50 Gy) followed by LE	CRT (50.4 Gy) followed by TME	Short- and long-term	36
Serra-Aracil 2023/2025 (Spain)	RCT (17)	81 vs. 81	T2-3 N0 AV ≤ 10 cm Size ≤4 cm	CRT (50.4 Gy) followed by TEM	Upfront TME	2023: Short-term 2025: Long-term	63

*UK *United Kingdom; *China *People’s Republic of China, *RCT *randomized controlled trial, *AV *anal verge, *cCR *clinical complete response, *TEM *transanal endoscopic microsurgery, *LE *local excision, *TME *total mesorectal excision, *SCRT *short-course radiotherapy, *CRT *chemoradiotherapy

**Table 2:** Details of clinical and pathological tumor stages

Author and year (country)	Clinical tumor stage	*n* (%)*	Pathological tumor stage	*n* (%)*
Bach 2021 (UK)	cT1N0 cT2N0	20 (23%) vs. 5 (14%) 61 (69%) vs. 28 (80%)	ypT0/pT0 ypT1/pT1 ypT2/pT2 ypT3/pT3	27 (31%) vs. 0 24 (27%) vs. 15 (43%) 20 (23%) vs. 19(54%) 9 (10%) vs. 1 (3%)
			pN0 pN1 pN2	NA vs. 30 (86%) NA vs. 3 (9%) NA vs. 1 (3%)
Lezoche 2008 (Italy)	cT2N0	35 (100%) vs. 35 (100%)	ypT0 ypT1 ypT2	11 (32%) vs. 10 (29%) 6 (17%) vs. 7 (20%) 18 (51%) vs. 18 (51%)
Lezoche 2012 (Italy)	cT2N0	50 (100%) vs. 50 (100%)	ypT0 ypT1 ypT2	14 (28%) vs. 13 (26%) 12 (24%) vs. 12 (24%) 24 (48%) vs. 25 (50%)
Qiu 2025 (China)	cT1 cT2 cT3 cT4	2 (5%) vs. 0 6 (16%) vs. 7 (17%) 30 (79%) vs. 33 (80%) 0 vs. 1 (2%)	ypT0 yPT1 ypT2 ypT3	22 (58%) vs. 21 (51%) 5 (13%) vs. 5 (12%) 10 (26%) vs. 13(32%) 1 (3%) vs. 2(5%)
	cN0 cN1 cN2	16 (42%) vs. 15 (37%) 15 (39%) vs. 17 (41%) 7 (18%) vs. 9 (22%)	ypN0 ypN1	NA vs. 37 (90%) NA vs. 4 (10%)
Rullier 2017/2020 (France)	cT2 cT3	41 (55%) vs. 36 (51%) 33 (45%) vs. 35 (49%)	ypT0 ypT1 ypT2 ypT3	57 (40%; total) 29 (20%; total) 44 (31%; total) 12 (9%; total)
	cN0 cN1	42 (57%) vs. 48 (68%) 32 (43%) vs. 23 (32%)		
Serra-Aracil 2023/2025 (Spain)	cT2N0 cT3N0	113 (70%) 49 (30%)	ypT0/pT0 ypT1/pT1 ypT2/pT2 ypT3/pT3	35 (44%) vs. 0 18 (23%) vs. 10 (12%) 20 (25%) vs. 53 (65%) 6 (8%) vs. 18 (22%)
			pN0 pN+	NA vs. 64 (79%) NA vs. 17 (21%)

*Values are presented as LE + RT vs. TME

*UK *United Kingdom, *China *People’s Republic of China, *NA *not applicable

### Bias and quality analysis

**Fig. 2:**
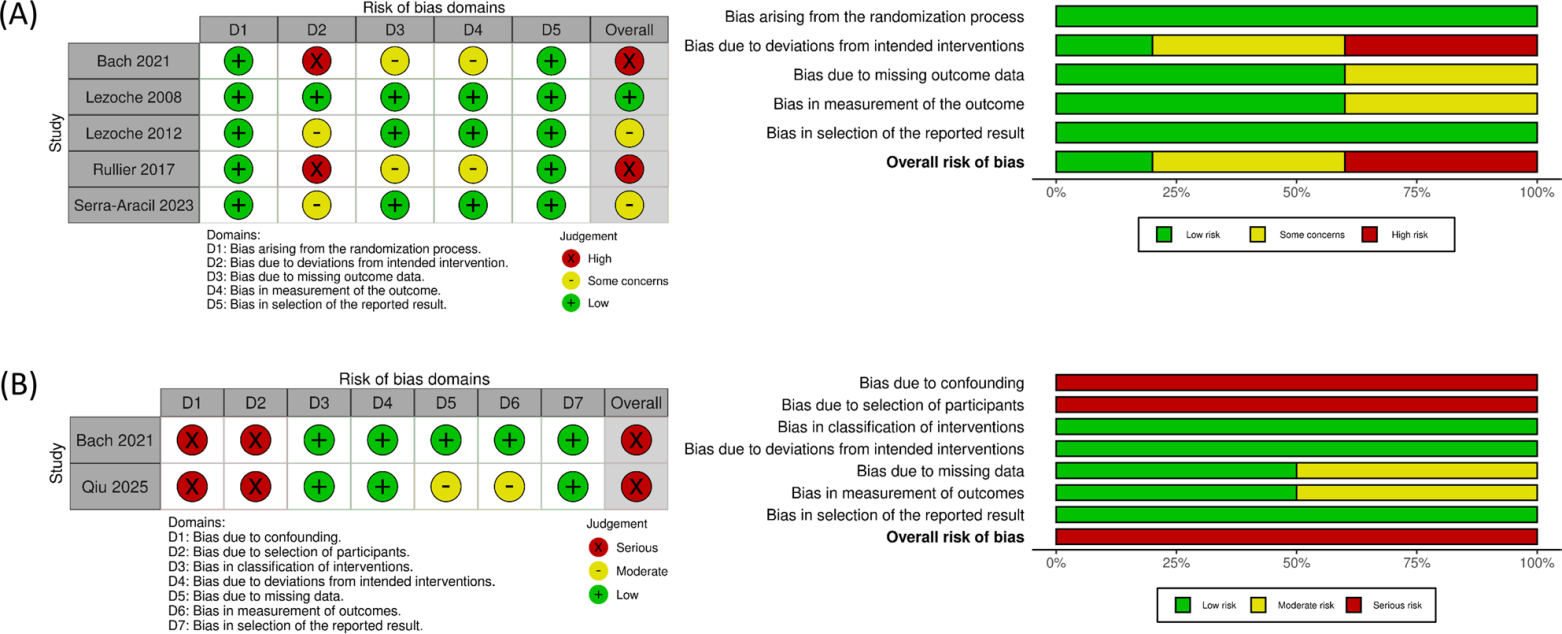
Bias and quality assessment of the included studies: (**A**) RoB 2; (**B**) ROBINS-I

The results of the risk of bias and quality assessments for all included studies, conducted using the RoB 2 and ROBINS-I tools, are presented in Fig. 2. In one RCT [24], both randomized and non-randomized prospective cohorts were reported. Accordingly, the risk of bias was assessed using the RoB 2 tool for the randomized cohort and the ROBINS-I tool for the non-randomized prospective cohort.

In the RCTs, blinding and allocation concealment were challenging to implement due to the surgical nature of the interventions. As a result, bias due to deviations from the intended intervention (Domain D2) could not be fully eliminated, and in two of the five RCTs, it was rated as high risk. Consequently, the overall risk of bias across the included RCTs ranged from low to high. However, even if deviations from the intended interventions occurred after randomization, this pooled analysis was based on the intention-to-treat (ITT) results reported in the individual trials.

In the non-randomized prospective cohorts extracted from two of the included studies, bias due to confounding factors and participant selection was judged to be serious, and the overall risk of bias was subsequently rated as serious. However, in both studies, patient selection with respect to the response to neoadjuvant therapy could not have occurred due to their study design.

### Postoperative outcomes

**Fig. 3:**
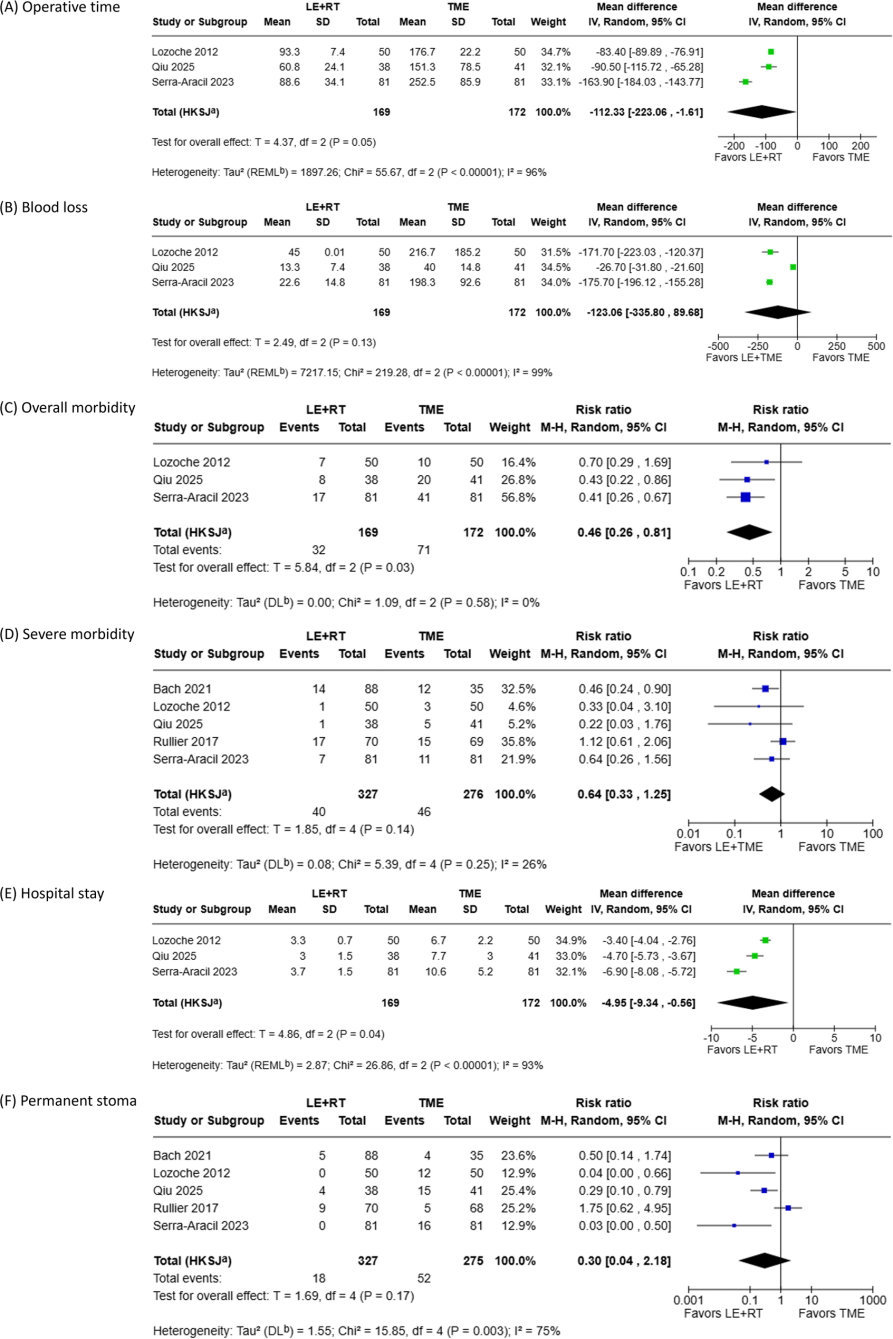
Meta-analyses of postoperative outcomes: (**A**) operative time; (**B**) blood loss; (**C**) risk of overall morbidity, including all Clavien–Dindo grades; (**D**) risk of severe morbidity (Clavien–Dindo grade ≥ III); (**E**) length of postoperative hospital stay; (**F**) risk of permanent stoma

Postoperative outcomes from the six included studies are summarized in Supplementary Tables 2, and the corresponding meta-analyses are presented in Fig. 3. Operative time, overall postoperative morbidity, and length of postoperative hospital stay were each analyzed using data from three studies comprising 341 patients (169 in the LE + RT group and 172 in the TME group).

The LE + RT group was associated with a significantly shorter operative time as compared to the TME group (MD: -112.33; 95% CI: -223.06 to -1.61; *p* = 0.05), although substantial heterogeneity was observed (*p* < 0.01, I² = 96%). The LE + RT group was also associated with a significantly lower risk of overall postoperative morbidity (RR: 0.46; 95% CI: 0.26–0.81; *p* = 0.03), with no evidence of heterogeneity (*p* = 0.58, I² = 0%). In addition, the LE + RT group was associated with a significantly shorter postoperative hospital stay (MD: -4.95; 95% CI: -9.34 to -0.56; *p* = 0.04), although substantial heterogeneity was present (*p* < 0.01, I² = 93%).

Severe postoperative morbidity, blood loss, and the risk of permanent stoma did not differ significantly between the LE + RT and TME groups.

### Oncological outcomes

**Fig. 4:**
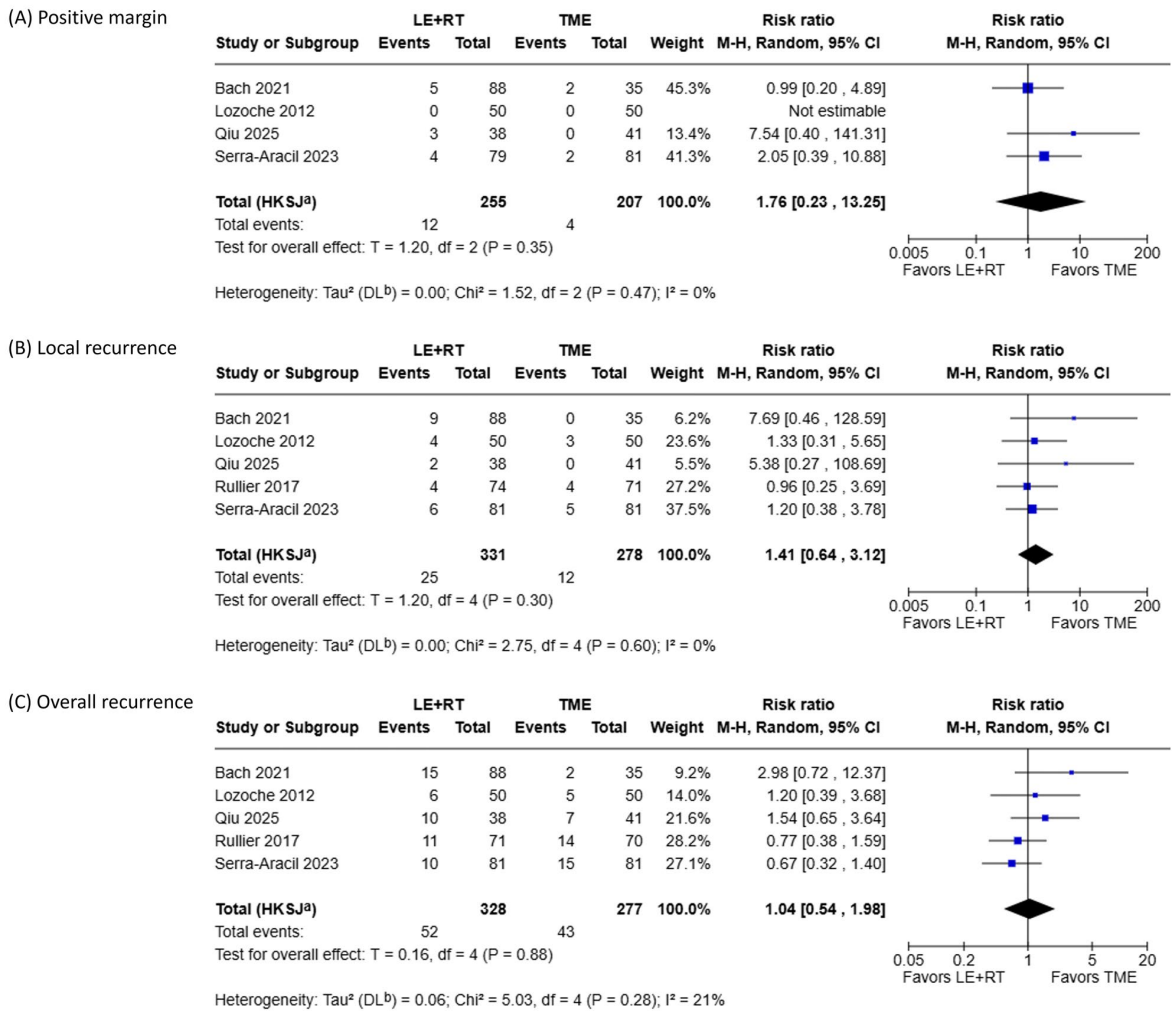
Meta-analyses of oncological outcomes: (**A**) risk of positive surgical margins; (**B**) risk of local recurrence; (**C**) risk of overall recurrence, including both local and distant metastases

Oncological outcomes from the six included studies are summarized in Table 3, and the corresponding meta-analyses are presented in Fig. 4. As shown in Fig. 4A, the LE + RT group tended to present a higher risk of positive surgical margins as compared to the TME group, although the difference was not statistically significant. No substantial heterogeneity was observed among studies.

**Table 3:** Summary of oncological outcomes extracted from the six included studies

Author and year (country)	Positive margin (*n*, %)*	Local recurrence (*n*, %)*	Distant recurrence (*n*, %)*	5-year OS (%)*	5-year-DFS (*n*, %)*
Bach 2021 (UK)	5 (6%) vs. 2 (6%)	9 (10%) vs. 0	9 (10%) vs. 2 (6%)	88 vs. 93^†^	76 vs. 85‡
Lezoche 2008 (Italy)	NR	2 (6%) vs. 1 (3%)	1 (3%) vs. 1 (3%)	NR	NR
Lezoche 2012 (Italy)	0 vs. 0	4 (8%) vs. 3 (6%)	2 (4%) vs. 2 (4%)	NR	NR
Qiu 2025 (China)	3 (8%) vs. 0	2 (5%) vs. 0	8 (21%) vs. 7 (17%)	93.2 vs. 88.2	75.6 vs. 80.9
Rullier 2020 (France)	NR	5 (7%) vs. 5 (7%)	13 (18%) vs. 13 (19%)	66 vs. 53	54 vs. 49
Serra-Aracil 2025 (Spain)	4 (5%) vs. 2 (2%)	6 (7%) vs. 5 (6%)	10 (12%) vs. 14 (17%)	82.7 vs. 85.2	88.9 vs. 88.9

*Values are presented as LE + RT vs. TME

† 3-year OS in the randomized cohort

‡ 3-year DFS in the randomized cohort

*UK *United Kingdom, *China *People’s Republic of China, *NR *not reported, *OS *overall survival, *DFS *disease-free survival, *LE *local excision, *RT *neoadjuvant radiotherapy, *TME *total mesorectal excision

The average median follow-up period across the six studies was 65.5 months. A meta-analysis of local and overall recurrence was performed using data from five studies comprising 609 patients (331 in the LE + RT group and 278 in the TME group). As shown in Fig. 4B, the LE + RT group tended to present a higher risk of local recurrence as compared to the TME group, although the difference was not statistically significant. As shown in Fig. 4C, the risk of overall recurrence did not differ significantly between the LE + RT and TME groups. Pooled analyses for both local and overall recurrence did not show any substantial heterogeneity.

### Survival Outcomes

**Fig. 5:**
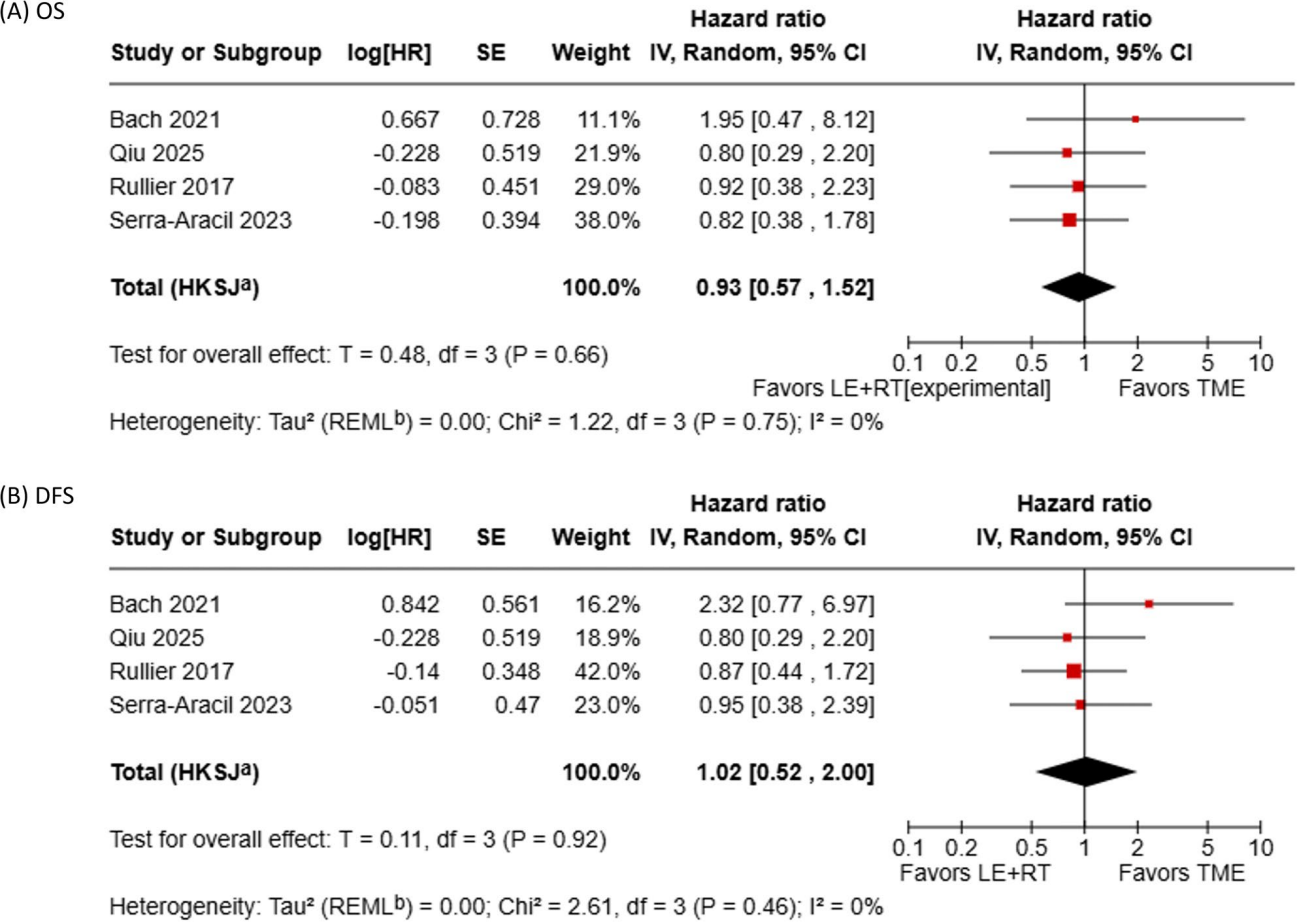
Meta-analyses of survival outcomes: (**A**) overall survival (OS); (**B**) disease-free survival (DFS)

Figure 5 shows the results of the meta-analyses for OS and DFS. A meta-analysis of survival outcomes was conducted using four studies comparing the LE + RT group with the TME group. In Fig. 5A, the pooled HR for OS was 0.93 (95% CI, 0.57–1.52; *p* = 0.66), indicating no significant difference between the LE + RT and TME groups. Heterogeneity among the studies was low (*p* = 0.75, I² = 0%). In Fig. 5B, the pooled HR for DFS was 1.02 (95% CI, 0.52–2.00; *p* = 0.92), similarly indicating no significant difference between the two groups. Heterogeneity was low (*p* = 0.46, I² = 0%).

## Discussion

This systematic review and meta-analysis showed that the LE + RT group was associated with a shorter operative time and a lower risk of postoperative complications as compared to the TME group. Additionally, the rates of local and overall recurrence, as well as OS and DFS, in the LE + RT group were comparable to those observed in the TME group. Consequently, neoadjuvant RT followed by LE may represent a viable alternative for selected patients with tumors up to stage cT3 and/or those showing a good response to neoadjuvant RT.

According to the previous version of the ESMO Clinical Practice Guidelines for rectal cancer published in 2017, LE was recommended as a treatment option only for very early cT1N0 tumors with low-grade (G1/G2) histology. The guidelines also described that RT/CRT followed by LE for cT2 tumors < 4 cm could be considered an alternative approach to TME, but it was not routinely recommended outside clinical trials, except for elderly or frail patients at high surgical risk [29]. In contrast, in the latest version of the ESMO Clinical Practice Guidelines for localized rectal cancer published in 2025, neoadjuvant RT followed by LE has become one of the management options when organ preservation is intended, particularly for patients with baseline cT2 or cT3aN0 tumors who achieve nCR [30]. As treatment options have expanded and become more complex, the selection of the optimal strategy, whether TME, LE, or WW, should be individualized based on tumor characteristics, treatment response, and patient factors. Although decision-making is increasingly individualized, LE can now be considered one of the viable options within a personalized treatment framework.

Initially, patients who achieved cCR were the only candidates for WW [8, 13]; However, pathological findings after TME in nCR patients revealed that approximately half of the cases were pCR [31, 32]. As a result, patients with nCR have also been considered for WW. As compared to the well-defined criteria for cCR, the criteria and characteristics for nCR vary substantially, and a systematic review of nCR definitions has highlighted wide variations in the scoring systems and terminology used for its description [33]. Additionally, the local regrowth rate in the nCR group was 52%, as compared to 22% in the cCR group [14]. In the present meta-analysis, the inclusion criteria of the included studies primarily consisted of cT3N0 disease with tumor size < 3 to 4 cm, representing patients with favorable tumor characteristics who were more likely to achieve cCR or nCR after neoadjuvant therapy. Although two studies allowed the inclusion of patients with cT4 or cN+ disease, LE was performed only in those who achieved cCR or nCR. In patients with cCR, WW has emerged as a non-operative management option; consequently, LE may be considered an alternative organ-preserving approach in selected patients with nCR. However, in patients with iCR, the role of LE compared with TME remains uncertain, and further prospective studies are warranted to establish its oncological safety and clinical benefit.

Since its introduction, TEM, which uses a rigid transanal endoscope, has become the treatment of choice for benign rectal lesions and early T1 rectal cancer not amenable to flexible endoscopic techniques such as endoscopic mucosal resection (EMR) or endoscopic submucosal dissection (ESD) [34]. Subsequently, transanal minimally invasive surgery (TAMIS), which incorporates the principles of TEM and uses a disposable soft transanal platform, has emerged as a viable alternative [35]. However, TEM for rectal cancer remains technically demanding, requiring substantial expertise and standardization due to its procedural complexity [36]. In our study, the difference was not statistically significant, the rate of positive surgical margins tended to be higher in the LE + RT group than in the TME group, underscoring the technical challenges in achieving an R0 resection. Specifically, following RT, treatment-induced fibrosis and inflammation can obscure the precise location and extent of the residual tumor scar. As a result, the widespread clinical adoption of such techniques should be addressed with caution.

Evidence in this field is still accumulating. A RCT is currently underway in Korea, comparing LE and TME in patients with rectal adenocarcinoma located < 8 cm from the anal verge who have undergone CRT [37]. Although only good responders to CRT are eligible, patients with baseline stages cT3-4N0 or cTanyN1-2 are also included, indicating that the results will provide insights into a more advanced disease cohort. Another ongoing RCT, the TESAR trial in the Netherlands, includes patients with high-risk pT1 or low-risk pT2 tumors [38]. This trial compares adjuvant CRT with additional TME after LE. Because LE is performed first, the trial does not restrict inclusion to good responders to CRT. Together, these trials are expected to broaden the role of LE in rectal cancer management.

Functional outcomes represent a critical consideration when comparing LE and TME, particularly in the context of organ-preserving strategies. However, in this meta-analysis, pooled analysis of functional and QoL outcomes was not feasible due to substantial heterogeneity in assessment tools and reporting methods, which represents an important limitation of the present study. Nevertheless, available evidence suggests potential functional advantages associated with LE. For instance, Qiu et al. reported significantly better postoperative Wexner scores and low anterior resection syndrome scores in the LE group [26]. Beyond anal function, genitourinary function and overall QoL are also major considerations. To enable meaningful comparisons, standardized evaluation methods and validated instruments should be consistently applied in future studies. Further accumulation of high-quality data in this field is warranted.

This study has several limitations. First, substantial heterogeneity was observed among included studies, likely reflecting clinical and methodological differences. These included variations in study design, as two of the included studies were non-randomized cohorts, as well as differences in treatment protocols, such as RT type (CRT vs. SCRT) and TME approach (upfront TME vs. CRT followed by TME). In addition, patient selection criteria varied across studies with respect to clinical stage, tumor size, tumor location from the anal verge, and the use of tumor regression after CRT as a selection factor. Consequently, the findings of this meta-analysis should be interpreted with caution and are most applicable to carefully selected patients with locally advanced rectal cancer, particularly those demonstrating a favorable response to neoadjuvant RT and considered suitable for organ-preserving strategies. In addition, patient selection criteria varied across studies with respect to clinical stage, tumor size, tumor location from the anal verge, and the use of tumor regression after CRT as a selection factor. Consequently, the findings of this meta-analysis should be interpreted with caution and are most applicable to carefully selected patients with locally advanced rectal cancer, particularly those demonstrating a favorable response to neoadjuvant RT and considered suitable for organ-preserving strategies. Second, regarding the risk of bias assessment, particularly in the two included RCTs, crossover from the LE + RT group to the TME group was observed, representing a deviation from the intended intervention. This crossover may have influenced the observed postoperative morbidity and oncological outcomes and could have attenuated the differences between treatment groups. Consequently, these findings should be interpreted with caution. Third, the relatively small sample sizes of the included studies may have contributed to instability of the pooled estimates. Finally, the generalizability of these findings may be limited, particularly for patients with cT3 tumors achieving nCR, due to the relatively small number of such cases included in the analysis. Additionally, the LE + RT group, although not statistically significant, showed a trend towards higher rates of positive margins and local recurrence in some studies, which should be interpreted with caution.

Despite such limitations, this study provides valuable insights and may serve as a major impetus for refining treatment strategies in selected patients, particularly those with intermediate-stage rectal cancer (e.g., cT2-cT3a/b, cN0) who achieve nCR after neoadjuvant therapy.

## Conclusions

This systematic review and meta-analysis showed that, in selected patients with rectal cancer, neoadjuvant RT followed by LE was associated with more favorable perioperative outcomes and comparable oncological and survival outcomes to TME. Local excision (LE) combined with neoadjuvant RT may represent a viable treatment option, particularly in patients who strongly desire organ preservation or who are unfit for TME. To facilitate individualized treatment strategies, LE may be appropriately integrated with existing approaches such as TME and the WW.

## Supplementary Information

Supplementary Material 1.

## Data Availability

The data are available from the corresponding author upon reasonable request.
