# Supplementary material for: Systematic review and meta-analysis comparing postoperative and oncological outcomes between local excision plus radiotherapy versus total mesorectal excision for rectal cancer
Source: World J Surg Oncol. 2026 Apr 2;24:203. doi: 10.1186/s12957-026-04304-8 (PMC13169656; doi:10.1186/s12957-026-04304-8)
Supplement: Supplementary file 1 — Supplementary Material 1. [file 12957_2026_4304_MOESM1_ESM.docx]

**Supplementary Table 1:** Search strategies for each database

| Database | Date of last search | Search strategy |
| --- | --- | --- |
| PubMed | 2025-04-30 | ("Rectal Neoplasms"[Mesh] OR rectal cancer[tiab] OR rectal neoplasm*[tiab] OR rectal tumor*[tiab]) AND (local excision[tiab] OR transanal excision[tiab] OR transanal endoscopic microsurgery[tiab] OR transanal minimally invasive surgery[tiab] OR TEM[tiab] OR TAMIS[tiab] OR "Transanal Endoscopic Microsurgery"[Mesh]) AND ("Rectal Neoplasms/surgery"[Mesh] OR "Proctectomy"[Mesh] OR total mesorectal excision[tiab] OR TME[tiab]) |
| Embase | 2025-04-30 | ('rectum tumor'/exp OR 'rectal cancer':ti,ab OR 'rectal neoplasm*':ti,ab) AND ('local excision'/exp OR 'transanal excision':ti,ab OR 'transanal endoscopic microsurgery':ti,ab OR TEM:ti,ab OR TAMIS:ti,ab) AND ('total mesorectal excision'/exp OR 'total mesorectal excision':ti,ab OR TME:ti,ab) |
| Cochrane Library | 2025-04-30 | (rectal neoplasms OR rectal cancer) AND (local excision OR transanal excision OR TEM OR TAMIS) AND (total mesorectal excision OR TME) |

**Supplementary Table 2:** Summary of postoperative outcomes extracted from the six included studies

| Author and year  (country) | Operative time  (min, mean)* | Blood loss  (mL, mean)* | Overall morbidity  (n, %)* | Severe morbidity  (n, %)* | Hospital stay  (days, mean)* | Permanent stoma  (n, %)* |
| --- | --- | --- | --- | --- | --- | --- |
| Bach 2021 (UK) |  |  |  | 14 (16%) vs. 12 (34%) |  | 5 (6%) vs. 4 (11%) |
| Lezoche 2008 (Italy) | 93.3 vs. 176.7 | 46.7 vs. 216.7 | 5 (14%) vs. 6 (17%) | 1 (3%) vs. 2 (6%) | 4.0 vs. 6.7 | 0 vs. 9 (26%) |
| Lezoche 2012 (Italy) | 93.3 vs. 174.7 | 45.0 vs. 216.6 | 7 (14%) vs. 10 (20%) | 1 (2%) vs. 3 (6%) | 3.3 vs. 6.0 | 0 vs. 12 (24%) |
| Qiu 2025 (China) | 60.8 vs. 151.3 | 13.3 vs. 40.0 | 8 (21%) vs. 20 (49%) | 1 (3%) vs. 5 (12%) | 3.0 vs. 7.7 | 4 (11%) vs. 15 (37%) |
| Rullier 2017 (France) |  |  |  | 17 (24%) vs. 15 (22%) |  | 9 (13%) vs. 5 (7%) |
| Serra-Aracil 2023 (Spain) | 88.6 vs. 252.5 | 22.6 vs. 198.3 | 17 (21%) vs. 41 (51%) | 7 (9%) vs. 11 (14%) | 3.7 vs. 10.6 | 0 vs. 16 (20%) |

*Values are presented as LE+RT vs. TME.

UK, United Kingdom; China, People’s Republic of China; LE, local excision; RT, neoadjuvant radiotherapy; TME, total mesorectal excision.
